# Moral courage and psychological empowerment among nurses

**DOI:** 10.1186/s12912-020-00435-9

**Published:** 2020-05-24

**Authors:** Zahra Khoshmehr, Maasoumeh Barkhordari-Sharifabad, Khadijeh Nasiriani, Hossein Fallahzadeh

**Affiliations:** 1 Department of Nursing, School of Medical Sciences, Yazd Branch, Islamic Azad University, Shohadaye Gomnam Blvd., Safaiyeh, Yazd, Postal code: 8916871967 Iran; 2 Department of Nursing, Nursing and Midwifery Research Center, Research Center for Neonate & Mother, Shahid Sadoughi University of Medical Sciences and Health Services, Yazd, Iran; 3 Department of Biostatistics and Epidemiology, Faculty of Health, Shahid Sadoughi University of Medical Sciences and Health Services, Yazd, Iran

**Keywords:** Ethics, Moral courage, Moral action, Psychological empowerment, Nurse

## Abstract

**Background:**

Moral courage is one of the fundamental values of nursing profession and a powerful method of coping with ethical problems. Psychological empowerment is a suitable method of enabling individuals to coping mental pressures of the work environment. This study determined the correlation between moral courage and psychological empowerment of nurses.

**Methods:**

This was a descriptive cross-sectional study. A total of 180 nurses employed in different wards were selected randomly. Data were collected by Demographics Questionnaire, Sekerka’s Moral Courage Scale, and Spreitzer’s psychological empowerment Scale and analyzed with SPSS16 using descriptive and inferential statistics.

**Findings:**

The results indicated that the mean score of moral courage was 21.11 ± 69.90 and the greatest amount of moral courage was in the dimension of “going beyond compliance”. The mean score of “psychological empowerment” was 30.9 ± 73.58 and the greatest mean belonged to “competence”. There was a positive significant correlation between “psychological empowerment” and “moral courage and its dimensions” (*P* < 0.05).

**Conclusion:**

The findings suggested a correlation between moral courage and psychological empowerment. Thus, nurses’ moral courage could be enhanced by reinforcing their psychological empowerment leading to increased patient satisfaction and quality care.

## Background

Morality is an indispensable part of human life and a subset of practical philosophy looking for the right and wrong and determining good and bad in a collection of behaviors under certain conditions [1]. The nursing profession is one of the sciences with abundant illustrative ethical aspects in the past, present, and future [2, 3]. Since distinguishing the good and bad is in the body of ethics, the moral competency of the nursing profession may be rendered as equal to professional competency [3].

Indeed, nurses face some moral problems in their daily work that need to be resolved [4, 5]. Identification of ethical problems requires moral sensitivity [6], and also an awareness of ethical principles [7]. Nonetheless, mere moral sensitivity and knowledge will not suffice. Nurses ought to possess moral courage to perform on the basis of what is considered ethically right provided personal values and criteria correspond to the accepted healthcare values [4].

When a person is not able to act according to the correct ethical performance, moral courage helps them to try their best to achieve their ultimate goal regardless of its consequences. To do so, they consider moral principles and perform a correct act that is not easy to do [8]. Some studies have demonstrated that moral courage is related to concepts concerning assessment of ethics under certain conditions like sensitivity to justice [9], perception of control on one’s emotions and performance such as emotional self-regulation [10], and self-efficacy [11]. Moral courage predisposes to performing ethical norms regardless of social costs [12], helping patients reduce symptoms of pain and agony, communicating effectively with patients and their families, and cooperating with physicians [13], inclination for recognizing others’ sufferings and sensitivity to them, expression of sympathy and kindness, helping the needy, doing something to decrease others’ pains and sufferings, and challenging the current situation [14].

Stress, anxiety, fear of being scolded, and rejection by colleagues and seclusion are some of the negative consequences that may be created by moral courage [15]. Moral courage helps nurses overcome many barriers like fear, thence enabling them to defend the patient effectively [16]. In their study, Day reported the rate of moral courage as low in healthcare settings and mentioned many factors such as lack of occupational certainty as barriers to nurses’ inclination for brave behaviors [17].

On the other hand, courage is the token of a powerful nurse’s good performance and quality care. Empowerment is a process completed by personal values and struggles and also by environmental factors [18]. Psychological empowerment is an appropriate solution for enabling individuals to cope with mental pressures and work stressors [19]. Studies have shown that psychological empowerment is correlated with professional satisfaction [20], exerting a predictive effect on emotional commitment [21]. The results of many studies have suggested that empowerment of personnel influences responsibility, productivity, and quality of care [22], diminishes costs, enhances organizational loyalty and confidence, and organizational entrepreneurship [23]. The findings of other studies also demonstrate that increased development and empowerment leads to reduced staff displacement, fatigue and work leave [24], promoted quality of nursing care [25], increased patient satisfaction [20], increased occupational satisfaction, and decreased turnover of nurses [26].

Observing professional ethics is an effective and powerful factor in nurses’ development [27]. Ethic of care, that is one component of nursing performance, creates and promotes moral courage [7]. Although moral courage is rendered as an important element of nursing, little attention has been paid to it so that there are very few studies focusing on this topic. Hence, this study investigated the correlation between nurses’ moral courage and psychological empowerment.

## Methods

### Design of the Study

This descriptive cross-sectional study was conducted in 2019. The study population consisted of all nurses employed in Khatam-al-Anbia Hospital and Shahid Beheshti Hospital affiliated to Shahid Sadoughi University of Medical Sciences, Yazd, Iran. A total of 180 participants were selected randomly using sample volume formula with confidence interval of 95%, test power of 80% and the correlation coefficient of 0.18 according to the pilot study. The inclusion criteria were: holding at least a Bachelor of Science (BS) in nursing, at least 1 year of clinical nursing experience, and inclination for participation. The research instruments were distributed by the researcher in various work shifts and collected after completion.

### Data collection instruments

Data were collected by Demographics Questionnaire, Sekerka’s Moral Courage Scale, and Spreitzer’s Psychological Empowerment Scale.

The Demographics Questionnaire included information on age, gender, employment status, literacy level, marital status, official position, and work experience.

The Moral Courage Scale was developed by Sekerka et al. [28]. This 15-item scale covers five aspects: moral agency, multiple values, endurance of threat, going beyond compliance, and moral goals. Each aspect includes 3 separate items. This instrument uses a 7-point Likert scale wherein each item receives 1–7 points (from never correct = 1 to always correct = 7). Thus, the score of each item may range from 3 to 21. The minimum and maximum total scores were 15 and 105, respectively. The mean score of items in each aspect and in the whole item was considered as the moral courage score [28]. The validity of Moral Courage Scale was reported as 81% in the study by Mohammadi et al. and its reliability was estimated to be 0.85 (Cronbach’s α) using a sample volume of 30 nurses under study [29].

Spreitzer’s Psychological Empowerment Scale was used to measure nurses’ psychological empowerment [30]. This 15-item inventory uses a 5-point Likert scale (form completely disagree = 1 to completely agree = 5) to measure 5 aspects: meaningfulness, competence, self-determination, impact, and confidence so that 3 items are devoted to each aspect. The scores of this tool ranged from 15 to 75 and a higher score indicated higher perceived psychological empowerment [30]. Content and face validities were confirmed using a qualitative method by asking the opinions of 10 expert professors. The reliability of the instrument was reported as Cronbach’s α = 0.84 [31].

### Data analysis

The gleaned data were imported to SPSS16 and analyzed with descriptive statistics (frequency distribution, mean and standard deviation) and inferential statistics (independent t-test, ANOVA, and Pearson correlation coefficient). Normality of data distribution was examined by Kolmogorov-Smirnov (KS) test (*P* > 0.05).

## Results

All 180 questionnaires were returned and analyzed. The mean age of the participants was 33.55 ± 6.07 years with a mean work experience of 2.40 ± 1.51 years. Most participants were female (79.4%), were married (81.1%), held a BS in nursing (93.9%), formally employed (61.7%), and held the post of a nurse (96.7%) (Table 1).

**Table 1:** Sociodemographic characteristics of study participant

Variables		M (SD)	***n*** (%)
Age		33.55 (6.07)	
Work Experience		2.40 (1.51)	
Gender	Female		143 (79.4)
	Male		37 (20.6)
Marital Status	Single		34 (18.9)
	Married		146 (81.1)
Level of Education	Bachelor’s Degree		169 (93.9)
	Master’s Degree		11 (6.1)
Employment status	Formal		111 (61.7)
	Compulsory service course		36 (20)
	By contract		33 (18.3)
Formal position	Nurse		174 (96.7)
	Supervisor and head nurse		6 (3.3)

The results revealed that nurses enjoy a high level of moral courage so that the greatest mean belonged to “going beyond compliance” and the least mean pertained to “multiple values”. Moreover, the findings demonstrated that the rate of nurses’ psychological empowerment was moderate so that the greatest mean belonged to “competence” and the smallest mean pertained to “confidence” (Table 2).

**Table 2:** Mean and SD of moral courage and psychological empowerment and its dimensions

Variable	Mean	SD
**Moral courage**	90.69	11.21
Moral agency	18.21	2.51
Multiple value	17.72	2.68
Endurance of threat	17.97	2.86
Going beyond compliance	18.48	2.31
Moral goal	18.30	2.58
**Psychological empowerment**	58.73	9.30
Competence	13.32	1.72
Self-determination	12.85	1.59
Impact	11.60	2.38
Meaning	11.35	2.35
Confidence	9.60	4.83

The findings indicated a positive significant correlation between “psychological empowerment” and “moral courage and its dimensions” (*P* < 0.05). Besides, there was a significant correlation between moral courage and all dimensions of psychological empowerment except for “confidence” (*P* < 0.05) (Table 3).

**Table 3:** Correlation between moral courage and its dimensions with psychological empowerment and its dimensions

Variables	Psychological empowerment		Competence		Self-determination		Impact		Meaning		Confidence	
	r	P	r	P	r	P	r	P	r	P	r	P
**Moral courage**	0.29	0.00^a^	0.22	0.002^a^	0.31	0.00^a^	0.26	0.00^a^	0.29	0.00^a^	0.10	0.16
**Moral agency**	0.23	0.002^a^	0.23	0.002^a^	0.24	0.001^a^	0.12	0.09	0.20	0.006^a^	0.12	0.10
**Multiple value**	0.36	0.00^a^	0.18	0.01^a^	0.28	0.00^a^	0.40	0.00^a^	0.37	0.00^a^	0.15	0.03^b^
**Endurance of threat**	0.19	0.009^a^	0.17	0.02^b^	0.23	0.001^a^	0.17	0.02^b^	0.19	0.008^a^	0.05	0.49
**Going beyond compliance**	0.21	0.004^a^	0.21	0.004^a^	0.33	0.00^a^	0.19	0.009^a^	0.01	0.83	0.23	0.001^a^
**Moral goal**	0.26	0.00^a^	0.19	0.009^a^	0.27	0.00^a^	0.22	0.002^a^	0.27	0.00^a^	0.09	0.19

^a^Correlation is significant at the 0.01 level (2-tailed)

^b^Correlation is significant at the 0.05 level (2-tailed)

The results of Pearson Correlation Coefficient Test showed a significant correlation between age and moral courage (*r* = 0.230, *P* = 0.002) and between work experience and moral courage (*r* = 0.181, *P* = 0.015) so that moral courage increased with increasing age and work experience (Table 4).

**Table 4:** Determining the correlation between moral courage and psychological empowerment scores and demographic variables

Variables			Moral courage	Psychological empowerment
Age		r	0.230	0.120
		P	0.002^a^	0.110
Work Experience		r	0.181	0.115
		P	0.015^b^	0.123
Gender	Female	Mean (SD)	90.58 (11.36)	58.86 (9.55)
	Male	Mean (SD)	91.10 (10.73)	58.24 (8.35)
		P	0.802	0.717
Marital Status	Single	Mean (SD)	87.88 (14.96)	57.32 (13.41)
	Married	Mean (SD)	91.34 (10.09)	59.06 (8.08)
		P	0.206	0.471
Level of Education	Bachelor’s Degree	Mean (SD)	90.57 (11.34)	58.68 (9.35)
	Master’s Degree	Mean (SD)	92.50 (6.28)	59.63 (8.81)
		P	0.689	0.724
Employment status	Formal	Mean (SD)	91.71 (9.66)	59.30 (7.94)
	Compulsory service course	Mean (SD)	88.97 (15.40)	57.58 (13.14)
	By contract	Mean (SD)	89.15 (10.65)	58.09 (8.66)
		P	0.305	0.572
Formal position	Nurse	Mean (SD)	90.63 (11.34)	58.60 (9.33)
	Supervisor and head nurse	Mean (SD)	92.50 (6.28)	62.50 (8.24)
		P	0.592	0.315

^a^Correlation is significant at the 0.01 level (2-tailed)

^b^Correlation is significant at the 0.05 level (2-tailed)

The rate of moral courage and psychological empowerment was higher among the married, those with MSc degree, head nurses/supervisors, and formally employed personnel. Moral courage score was greater in males whereas psychological empowerment score was higher in females, albeit, the difference was not statistically significant (*P* > 0.05) (Table 4).

## Discussion

This study determined the correlation between nurses’ moral courage and psychological empowerment. The findings showed that the participating nurses had a high degree of moral courage. This is consistent with the results of the study by Mahdaviseresht et al. [8], that reported a high mean score of nurses’ moral courage (90.36 ± 10.56). The studies by Taraz et al. [32], and Moosavi et al. [33], also reported a high rate of moral courage among nurses. Nonetheless, the study by Mohammadi et al. [29], reported nurses’ moral courage at the moderate level. In Day’s study, the rate of this variable was low [17]. The diversity of results in different studies may be attributed to disparities in work environment, ethical atmosphere, organizational culture, organizational and managerial support, fear of social seclusion, collective thought, and lack of acceptance by the organization [34].

In the present study, the highest score of moral courage belonged to “going beyond compliance” and the lowest score pertained to “multiple values”. Some studies reported the greatest amount of moral courage in “moral agency” [8, 32, 34]. This is not consistent with our results. Yet, consistent with our findings, in the studies by Mahdaviseresht et al. [8], and Taraz et al. [32], the lowest score of this variable pertained to “multiple values”. Nonetheless, in the study by Aminizadeh et al. [34], the lowest score of moral courage belonged to “endurance of threat”.

The high score of “going beyond compliance” indicates that nurses do not merely consider laws and regulations; rather, they progress beyond their internal capacity and consider doing what is correct and ideal [28, 35], suggesting nurses’ responsibility for their profession and patients. The low score of “multiple values” indicates nurses’ inability in ethical decision-making and coping with organizational constraints that may predispose to their moral apprehension, finally affecting their quality of care [28, 32, 35]. This needs to be noted by the responsible authorities. Also organizations must accept moral virtues such as courage and direct organizational culture towards supporting the nurses with moral courage. Some of the consequences are the right decision making, the right action, the patient’s safety and comfort, and playing the role of care [16].

The findings of the present study revealed that the mean score of nurses’ psychological empowerment was at the moderate level. The results of other studies are consistent with this finding [36–40]. Ouyang et al. reported in their study the Chinese nurses’ psychological empowerment at the moderate level [38]. Moreover, another study in Egypt, suggested a moderate level of nurses’ psychological empowerment [37]. Furthermore, the findings of a systematic review demonstrated that Iranian nurses’ psychological empowerment is moderate [39]. Yet, the study by Mirkamli et al. reported Iranian nurses’ psychological empowerment at a high level [41].

In the present study, the highest score of psychological empowerment pertained to “competence” whereas the lowest score belonged to “confidence”. In the study by Zahednezhad et al., the highest mean score belonged to “competence” while the lowest mean score pertained to “self-determination” [31], which is consistent with our study in maximal score. In the study by Mirkamli et al., the maximal mean pertained to “meaningfulness” whereas the minimal score belonged to “confidence” [41], that is consistent with our study in the minimal dimension.

The low score of nurses’ “confidence” in our study indicates that they are not sure that the powerful authorities will behave justly, honestly, and equally towards them; in other words, they do not feel any personal safety and security. Hence, nursing managers ought to pay due attention to this issue.

In our study, there was a direct significant correlation between psychological empowerment and its dimensions so that increased psychological empowerment resulted in nurses’ enhanced moral courage. No study was found to have exactly investigated the association between these two variables though some studies have indirectly implied it. For instance, LaSala et al. emphasize that all nurses in all roles and all work environments ought to commit themselves to creating a structurally powerful environment that supports moral courage [7]. ZahedBabelan et al. also revealed that moral behavior, with its high psychological empowerment path coefficients, can be considered as an influential and powerful factor in empowering or developing nurses [27]. Furthermore, Sadooghiasl et al. refer to ethical and scientific competence, self-construction, and rationalism as antecedents of moral courage. They also refer to protective environment and ethical climate of the organization as a factor contributing to moral courage [16]. Kuokkanen et al. describe courage, tenacity, and self-esteem as qualities of an empowered nurse. An empowered nurse is able to act under pressure, resist criticism, and act in their performance and professional positioning without any fear. Such a nurse accepts responsibility with courage in decision-making [18].

To clarify this finding, it may be said that since increasing psychological empowerment may lead to reduced mental pressures and work environment stressors, and enhance the power of decision-making and performing moral behavior by the nursing staff [42–44], it can ultimately result in the creation of moral courage in nurses. Having a sense of control, competency, autonomy, positive impact, and increased motivation in relation to work affects the moral courage of nurses. In these circumstances, nurses, as moral agency, can properly manage moral dilemmas. This will improve the quality of care.

The findings showed no significant correlation among demographic variables, moral courage and psychological empowerment except that moral courage was significantly promoted with increasing age and work experience. The study by Mohammadi et al. [29], and Moosavi et al. [33], further demonstrated a positive significant correlation between moral courage and work experience that is consistent with our study. With increasing age, individuals’ awareness of a situation is enhanced and their capacity for recognizing behaviors [45]. Aultman believes that moral courage is learned over time by observing the courageous behaviors of others. The occurrence of courageous behaviors is enhanced by increasing work experience and frequent encounters with therapeutic challenges [46]. Murray also states that as the work experience of nurses increases, the impact of barriers in the work environment on nurses’ performance decreases and moral courage increases [5]. Generally, moral courage and the decisive behaviors of nurses are enhanced with increasing age and work experience and familiarity with the work environment. Using experienced nurses as role modeling for junior nurses can be an effective factor in promoting nurses’ courageous behaviors.

### Limitations of the study

This study used self-reporting instruments for data collection. These instruments suffer from possibility of respondents’ fatigue and impatience in responding or shortage of time. Thus, some nurses may have not provided real answers. Another limitation was lack of control over intervening variables such as factors affecting personnel’s concentration that might have confounded the results.

## Conclusion

Given the correlation found between psychological empowerment and moral courage, it may be concluded that promotion of nurses’ psychological empowerment can increase their moral courage. Consequently, organizations and nursing managers are obliged to provide some strategies like changing managerial style in clinical wards, nurses’ contribution to decision-makings, and expanding a suitable organizational culture to move towards promoting nurses’ mental power and its various aspects as far as possible. Providing the necessary prerequisites for promoting nurses’ psychological empowerment can lead to increased morally courageous behaviors, ultimately ending in improved nursing quality care.

## Data Availability

The datasets generated and analyzed during the current study are not publicly available due to an agreement with the participants on the confidentiality of the data but are available from the corresponding author on reasonable request.

## Abbreviations

MSc: Master of science
BS: Bachelor of science
SD: Standard deviation
ANOVA: Analysis of variance
KS: Kolmogorov-Smirnov
